# Glow up: does a professional photoshoot intervention affect self-esteem and emotions among adolescent psychiatric patients?—A longitudinal intervention study

**DOI:** 10.3389/fpsyt.2024.1310252

**Published:** 2024-02-23

**Authors:** Kornelius Winds, Theresa Marka, Bernhard Salcher, Nicole Rieser, Christine Skrivanek, Michelle Hochrainer, Julia Trost-Schrems, Lucas J. Rainer, Wolfgang Hitzl, Christoph Augner, Belinda Plattner

**Affiliations:** ^1^ University Clinics for Child and Adolescent Psychiatry and Psychotherapeutic Medicine, Salzburger Landeskliniken, Paracelsus Medical University, Salzburg, Austria; ^2^ Centre for Cognitive Neuroscience Salzburg, Paris Lodron University Salzburg, Salzburg, Austria; ^3^ Department of Environment and Biodiversity, Paris Lodron University Salzburg, Salzburg, Austria; ^4^ University Clinics for Psychiatry, Psychotherapy and Psychosomatics, Salzburger Landeskliniken, Paracelsus Medical University, Salzburg, Austria; ^5^ University Clinics for Pediatrics, Paracelsus Medical University, Salzburg, Austria; ^6^ University Clinics for Neurology, Salzburger Landeskliniken, Paracelsus Medical University, Salzburg, Austria; ^7^ Research Office Biostatistics, Paracelsus Medical University, Salzburg, Austria; ^8^ Institute for Human Resources Research in Health Care, Salzburger Landeskliniken, Paracelsus Medical University, Salzburg, Austria

**Keywords:** children and adolescent psychiatry, self-esteem, state emotions, photo intervention, gender differences, social media, social networking sites

## Abstract

**Background:**

Today, online communication is shaped by a billion-dollar social media (SM) and social networking site (SNS) industry. Visual content consumed by children and adolescents has been shown to influence behavioral patterns, state emotions, and self-esteem (SE). In this study, we introduced a novel intervention creating visual content through a professional photoshoot and investigated its impact on state emotions and SE in child and adolescent psychiatric (CAP) patients.

**Methods:**

Standardized and validated self-rating questionnaires were used to assess SE, state emotions, coping mechanisms, psychopathological symptoms, and internet use behavior at baseline. SE and state emotions were monitored at different time points around a professional photoshoot within 45 CAP patients (30 female patients; mean age, 15.1 years) using a longitudinal design.

**Results:**

Within-subject repeated-measures ANOVA and bootstrapped paired-sample *t*-tests showed a significant fluctuation in state emotions and SE throughout the intervention. Spearman correlations and univariate logistic regressions revealed that internalizing symptomatology and maladaptive coping significantly worsened the outcome of the intervention on state emotions and SE in girls. Internet-related variables heightened the positive effect of the intervention in boys and lowered SE in girls during the intervention.

**Conclusion:**

The photo intervention had various gender-specific effects. Boys did benefit from the intervention in terms of longitudinal outcome on positive state emotions (PE) and SE, even positively influenced by SNS and SM. Thus, it might be concluded that online social comparison was processed more beneficial in boys. In contrast, when working with visual content in girls, psychopathology and coping must be considered. Internet consumption in general, especially SM and SNS, was related to low SE in girls. Nevertheless, when therapeutically accompanied, the “glow up moment” during the shoot (high on PE and SE; low on negative state emotions) could be used as an index moment for therapeutic reflection.

## 1 Introduction

With over five billion users worldwide, the internet plays a key role in the way we communicate today (1). Social media (SM) and social networking sites (SNS) have been found to promote pro-social functions, such as social support, friendship enhancement and maintenance, and to decrease loneliness (2).

In a parallel vein, SM and SNS are linked to a billion-dollar industry (3). The source of income for SM and SNS companies is product placement aiming to influence consumption behavior (4). Product presentation in SMS and SNS is characterized by behavior-relevant visual triggers, by communication through apparently like-minded peers (influencers), and by purchase recommendation with an ostensible non-commercial interest (5–9). The industry and its framework are accompanied by high-scale research on reachability of the main target group, which are young people in the phase of self-reflective identity seeking (10, 11). Products are presented by generally attractive, successful people of young age, leading to a continuous confrontation with perfect images of peers (12). Recently, an article in the *Wall Street Journal* leaked internal investigation results indicating that the use of Instagram increased body image dissatisfaction in adolescents, especially girls, leading to the development of eating disorders and depressions (13). Negative body image is associated with dissatisfied self-image and poses a high risk of developing low self-esteem (SE) (14), depression, and poor quality of life (14, 15). The irritation caused by idealized images on SM and SNS might be intentional, triggering the need to consume in order to adapt or to create an own desirable online personality (16).

SM and SNS aim at the basic human urge to communicate (17) and the human desire to be recognized by others (18). Needs such as visibility, acceptance, belonging, and at the same time recreational distraction are particularly present in adolescence (12, 19, 20), a phase characterized by various biological (21), psychological, and social changes (22) and affective regulation and identity formation processes (23). In particular, adolescents with low SE are more prone to instant messaging addiction (24) as well as problematic internet use (PIU) (25), while high SE may play a protective role in terms of PIU (26). PIU in adolescents can intensify maladjustment leading to altered self-concept and low self-satisfaction (27, 28), psychopathological symptoms, distinctive personality traits (29, 30), and problematic behavior among users (31, 32).

As psychopathological symptoms are often associated with poor SE and a negative self-perception (28, 33), creating a positive self-image is a major goal in psychotherapy with children and adolescents. Classic psychotherapeutic approaches are mainly language based and low SE is difficult to target, especially when it gets to personal appearance and body image. Being aware of the importance of visual content experiences online when it comes to social comparison and the impact on self-perception and identity development, an exclusively verbally conveyed approach seems insufficient when working with adolescent patients. Using photography as a therapeutic tool for psychiatric patients has been found to improve social skills, impulse control, and SE (34, 35). Different approaches such as the use of personal photographs as a representation for self-analysis (36, 37) or taking and posting a daily photo on SNS have been found to be related to positive therapeutic outcomes in terms of social interaction and emotion regulation (38–42).

Inspired by the project “Glow-up” created by Theresa Marka, in which she offered a professional photoshoot (PS) to her university fellows, we designed a study implementing a PS for child and adolescent psychiatric (CAP) patients (https://textmarka.com/category/project-glow-up/accessed on 05.10.2023). We chose a multiple time point assessment design in order to monitor the whole process including the phase of expectation, the actual shooting process, the reaction to the photos, and a follow-up in terms of SE and state emotions. We then analyzed the impact of psychopathological symptoms, coping strategies, and internet behavior on the course of the interventional outcome in CAP patients.

## 2 Methods, materials, and statistics

### 2.1 Participants and procedure

The study took place at the University Clinics for Child and Adolescent Psychiatry and Psychotherapeutic Medicine, Salzburger Landeskliniken, Paracelsus Medical University between June 2021 and February 2022. The department offers inpatient, outpatient, and day-clinic treatment and is the major supply hospital in the state of Salzburg, Austria.

A total sample of 45 children and adolescents was enrolled in the study. Inclusion criteria were (1) a minimum age of 12 (2), a maximum age of 20 (3), being a patient at University Clinics for Child and Adolescent Psychiatry and Psychotherapeutic Medicine, and (4) a written declaration of consent signed by patients and caregivers. Exclusion criteria were (1) inability to fill out self-rating questionnaires due to intellectual disability or insufficient German language proficiency (2), acute suicidality, and (3) eating disorder or acute severe psychiatric disorders with thought disorder and loss of reality (such as psychotic disorders and complex posttraumatic stress disorders) present at the time of the study. Inclusion and exclusion criteria were clinically assessed by the clinician responsible for psychiatric treatment.

**Figure 1:**
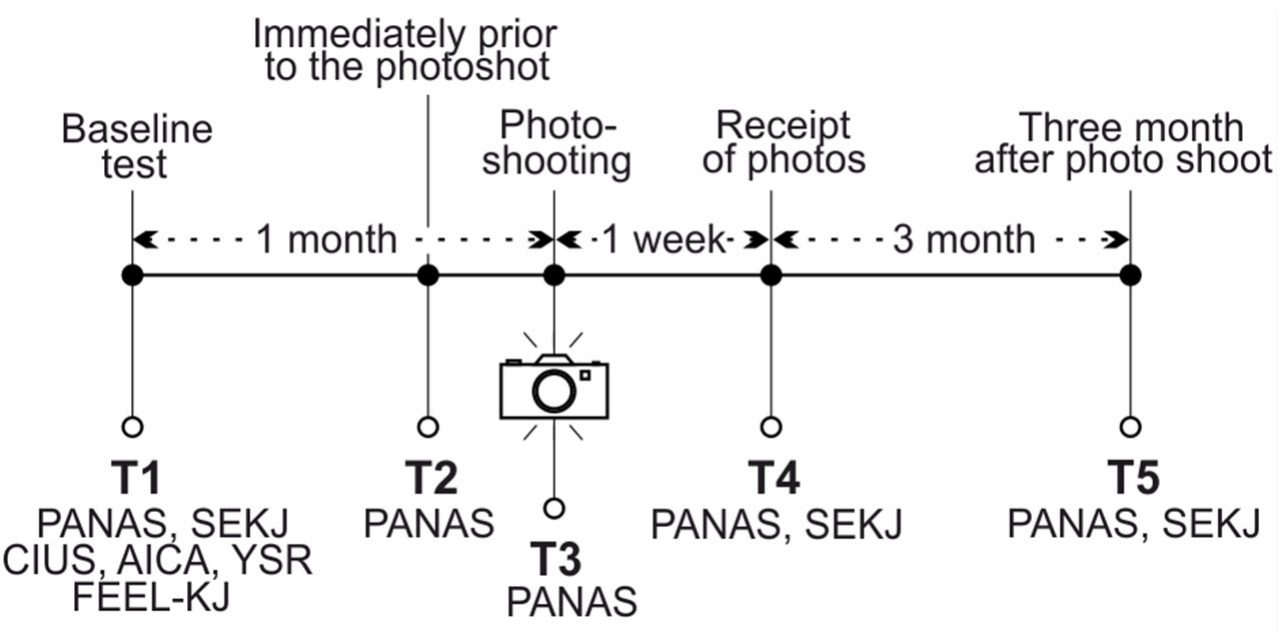
Test application in the longitudinal study design.

The same intervention procedure was chosen for each participant. After the invitation to participate in the study and consent, a baseline test was collected, further test points were immediately determined before the PS, during the PS, upon receipt of the pictures, and 3 months after receipt of the pictures. This made it possible to record emotions and SE in detail throughout the entire process (see **Figure 1**).

The PS took place in urban locations within walking distance from the clinics. Led by a professional photographer (Theresa Marka), the session spanned between 1.5 and 2 h, allowing participants ample time to feel at ease in front of the camera. The photography equipment utilized included a Canon 5D Mark II camera body with EF 24–70 mm f/2.8l and EF 70–200 mm f/2.8l lenses, set to program auto mode. Prior to the PS, the participants received a concise introduction to camera handling. The photographer conducted the PS individually with the participants, initiating the session with a stroll outside the clinic while engaging in light conversation to establish a sense of comfort and safety. The three designated photo locations were carefully selected to offer distinct settings. These included a serene nature spot, an urban locale adorned with vibrant graffiti capturing the essence of youth, and a backdrop featuring an architectural urban building. At the onset of the shoot, the photographer provided guidance on posing to help participants relax, subsequently minimizing interference to preserve authenticity. Employing professional techniques, diverse perspectives, including close-ups and long shots, wide-angle and portrait compositions, and full-body shots and shots from varying angles such as low and high, to capture a comprehensive view of the participants were incorporated during the PS. Post-PS, the pictures were imported to the photographer’s laptop and underwent minimal alteration, limited to color correction using the Adobe Lightroom application to achieve a more accurate representation of the scene, focusing on white balance adjustment, exposure and contrast, saturation and vibrance, hue adjustment, and tonal curve adjustments. This process was used to maintain a natural and authentic representation of the participants while ensuring that the colors were balanced. After the color correction, the pictures were exported as JPEGs onto a USB flash drive.

Approximately 1 week after the PS, each participant received between 120 and 150 photos on a personal USB flash drive. This procedure of receiving the photos was accompanied by one of the authors of the study and the photographer. The photos were shown to each participant and they were given opportunities to talk about the photos and their perceptions. After their talk, they completed the questionnaires of T4 (see **Figure 1**). To ensure privacy, the pictures were deleted from all data storage devices used during the procedure after handing out the USB flash drive. The participants were allowed to do whatever they want with the photos. There was no obligation to post the photos online.

### 2.2 Measures

#### 2.2.1 Positive and negative affect schedule

The Positive and Negative Affect Schedule (PANAS) is widely used to assess emotions (43, 44). It consists of 20 adjectives, which correlate to certain sensations and feelings. Items are rated on a five-point Likert scale ranging from 1 (= not at all) to 5 (= extremely). Summary scales for positive state emotions (PE) and negative state emotions (NE) are summarized based on 10 items, respectively (44). Depending on instruction, it is possible to measure affect in different contexts such as (i) the present moment, (ii) any past moment (day, week, or year), or (iii) in general (on average). The scale provides information to measure emotional responses to events as well as emotional fluctuations throughout a specific time frame (44). It is reliable and valid and shows a high internal consistency with a stability at appropriate levels over a period of 2 months (44). The German version of the PANAS has been derived from the English version (43).

#### 2.2.2 Self-esteem inventory for children and adolescents (German: Selbstwertinventar für Kinder und Jugendliche)

The Self-Esteem inventory for Children and Adolescents (SEKJ) contains three scales to assess important domains of SE, namely, height, stability over time, and academic contingency bound (45). All scales consist of 10 to 12 items/statements (32 items in total) scoring on a five-point rating scale to assess the extent of either agreement or disagreement. The internal consistency for all three scales scores between Cronbach α = 0.81 and 0.86 for 10- to 12-year-olds and between Cronbach α = 0.87 and 0.90 for 13- to 16-year-olds (46). The formulations of the specific scale items are based on systematic construct definitions and guarantee item validity of the inventory and were confirmed with two factor analyses (45). Expectation conform scale intercorrelations as well as associations with other factors such as depression, school phobia, rumination, and self-concept provide clear evidence for convergent/discriminant validity of the scales. The SEKJ was standardized in a sample of *n* = 3.121 pupils aged 10–16 years (45). The norm sample was representative for this age group in Germany in terms of geographical, gender, and school-type distribution (45).

#### 2.2.3 Compulsive internet use scale and scale for the assessment of internet and computer game addiction

We used a combination of the Compulsive Internet Use Scale (CIUS) (47) and the Scale for the Assessment of Internet and Computer Game Addiction (AICA) (48) to assess internet consumption behavior. The CIUS is a widely used self-report questionnaire that contains 14 items to assess internet use and its compulsiveness (47). The items measure loss of control, preoccupation (mental and behavioral), conflict (intrapersonal and interpersonal), withdrawal symptoms, and coping or mood modification on a five-point Likert scale. The CIUS sum score ranges from 0 to 56 points (47). A cutoff score of 21 points identifies a subthreshold PIU, whereas 28 points confirm PIU (49). The items of the CIUS are based on the Diagnostic and Statistical Manual of Mental Disorders version 4 criteria for dependence, obsessive–compulsive disorder, and behavioral addictions (47). The CIUS shows good reliability (Cronbach α = 0.89–0.90), good internal consistency (Cronbach α = 0.87), and good validity (47). The AICA was developed to assess the use of online formats (gaming, streaming, SM, and SNS) and appears in different versions (48). The screening version is a self-questionnaire tool based on the Diagnostic and Statistical Manual of Mental Disorders version 5 criteria for internet gaming disorder (50), consisting of six items measuring specific internet use within the last 12 months (51). When compared to external ratings of psychotherapists, scores of the self-report version of the AICA obtained a good diagnostic accuracy (sensitivity = 80,5%; specificity = 82,4%) as well as sound psychometric properties (52).

#### 2.2.4 Youth self-report

The Youth Self-Report (YSR) is a widely used self-report instrument, measuring behavioral and emotional problems among youth aged 11–18 years during the past 6 months (53). A total of 118 items can be rated on a three-point scale from 0 (= not true) to 2 (= very true). The scores lead to a total problem scale, two broadband scales (internalizing and externalizing problems) and eight empirically derived first-order syndrome scales (somatic complaints, social withdrawal, thought problems, social problems, anxiety/depression, aggressive behaviors, delinquent behaviors, and attention problems) (53). The YSR shows good validity, reliability, and internal consistency (54).

#### 2.2.5 Questionnaire for the evaluation of emotional regulation in children and adolescents (German: Fragebogen zur Erhebung der Emotionsregulation bei Kindern und Jugendlichen)

The Questionnaire for the evaluation of emotional regulation in children and adolescents (FEEL-KJ) is a standardized self-report questionnaire to assess emotion regulation (55). It contains 15 different strategies of emotion regulation consisting of five maladaptive, seven adaptive, and three other strategies. They are rated on five-point Likert scales evaluating the emotions sadness, fear, and anger. The questionnaire shows an internal consistency between Cronbach α = 0.69 and 0.93 (55).

#### 2.2.6 Narrative follow-up assessment

As part of the follow-up, the following questions were assessed based on written narrative. Question 1: How do you feel about the photos today? Question 2: Do you sometimes look at them? Question 3: Have you posted them on the Internet? For analyses, question 1 was coded into three categories: positive, ambivalent, and negative feelings toward the photos; question 2 was coded into two categories: yes/no revising the photos; and question 3 was coded into two categories: yes/no posting the photos.

### 2.3 Statistical and data analysis

Differences in demographics, internalizing and externalizing problems (YSR), maladaptive and adaptive strategies (FEEL-KJ), height of SE (SEKJ), PE and NE (PANAS), general internet, and specific internet behaviors (CIUS and AICA) between girls and boys were analyzed using chi-square tests for categorial variables and Mann–Whitney *U*-tests for continuous variables. Effect sizes of significant findings in chi-square tests were calculated using Cramers *V* with values >0.10 interpreted as small effects, >0.30 interpreted as medium effects, and 0.50 interpreted as large effects (56). Effect sizes of significant Mann–Whitney *U*-test findings were calculated using Cohen’s *d* with values >0.20 interpreted as small effects, >0.50 interpreted as medium effects, and 0.80 interpreted as large effects (56).

To test for the difference in mean scores of variables assessed in repeated observations, a within-subject repeated-measures ANOVA was performed using SPSS Version 28. Effect sizes were calculated using Cohen’s *d* (56). To test potential differences between the levels of PE, NE (PANAS), and SE (SEKJ) over time, we used bootstrapped paired-sample *t-*tests. For those sections in the interventional course that showed a significant rise or decline, simple linear regressions were administered to test if individual variables [internalizing and externalizing problems (YSR), maladaptive and adaptive strategies (FEEL-KJ), and general and specific internet use (CIUS and AICA)] significantly influence the section history. To assess possible influences of internet use-related variables, internalizing and externalizing symptomatology, and adaptive and maladaptive strategies on PE, NE, and SE at the different time points of the intervention, we performed two-tailed Spearman correlations.

All analyses were conducted in SPSS version 28.

### 2.4 Ethics

This study was approved on the 25 June 2021 by the ethics committee (ethic committee vote number: 1,091/2,021) of the state of Salzburg and was performed according to the Declaration of Helsinki 1995 (as revised in Edinburgh in 2000). All participants and their legal custodians provided written informed consent prior to participating in the study.

## 3 Results

### 3.1 Descriptive measures

Our study population consisted of 45 patients with a mean age of 15.51 years, of which *n* = 30 (66.7%) were female and *n* = 15 (33.3%) were male. A total of 24 participants received in-patient treatment, 13 received outpatient treatment, and 8 received day-clinic treatment.

Mean average use of the internet was 5.35 h/day on weekdays and 7.08 h/day on weekends. There was no difference in gender regarding the internet use on weekdays (*U* = 197.5, *Z* = −0.317, *p* = 0.751) or weekends (*U* = 174.5, *Z* = −0.899, *p* = 0.369). Psychopathology was obtained from our internal medical records. In the study, patients with diagnoses from the following categories were included: substance use disorders (*n* = 1), affective disorders (*n* = 3), neurotic, stress-related, and somatoform disorders (*n* = 23), personality disorders (*n* = 7), and disruptive behavior disorders (*n* = 11). There were no significant gender differences obtained, except for disruptive behavior disorders (male individuals, *n* = 7) [*χ* ^2^ (1, 45) = 6.016, *p* = 0.026].

Descriptive findings on sample characteristics and the results gained for internet behavior, psychopathological symptoms, adaptive/maladaptive strategies, PE, NE, and SE are shown in **Table 1** for each gender separately. Noteworthy, 46.7% of the girls and 80% of the boys showed subthreshold or full PIU. There were no significant differences in internet application use between the genders, respectively; 44.4% of our sample reported adaptive strategies below norm, and 73.3% reported maladaptive strategies above norm; 65.9% of our sample presented with SE below norm at T1. Significant differences between adaptive strategies within norm between girls and boys were present (*p* = 0.029).

**Table 1:** Descriptives for age and outcome measures of instruments used: CIUS (generalized internet behavior), AICA (application-related internet behavior), YSR (internalizing and externalizing problems), FEEL-KJ (coping strategies), PANAS (positive and negative state emotions), and SEKJ (Self-esteem).

	Total sample (*n* = 45)	Girls (*n* = 30)	Boys (*n* = 15)	Test^1,2^	Effect size^3,4^
Demographics					
Age (m, SD)	15.51 (1.66)	15.33 (1.647)	15.87 (1.685)	*p* = 0.249^1^	0.172^4^
CIUS					
CIUS score (m, SD)	23.80 (10.05)	22.20 (9.77)	27.00 (10.17)	*p* = 0.071^1^	0.269^4^
CIUS no PIU (CIUS ≤ 20; *n*, %)	19 (42.2)	16 (53.3)	3 (20.0)	*p* = 0.054^2^	0.318^3^
CIUS subthreshold PIU (CIUS = 21–27; *n*, %)	14 (31.1)	8 (26.7)	6 (40.0)	*p* = 0.497^2^	0.136^3^
CIUS PIU (CIUS ≥ 28; *n*, %)	12 (26.7)	6 (20.0)	6 (40.0)	*p* = 0.174^2^	0.213^3^
AICA categories					
AICA CP score (m, SD)	5.22 (6.47)	3.97 (6.40)	7.73 (6.04)	*p* = 0.015^1^	0.363^4^
AICA stream score (m, SD)	5.93 (5.77)	5.07 (5.47)	7.67 (6.14)	*p* = 0.150^1^	0.214^4^
AICA SM score (m, SD)	7.16 (6.15)	7.17 (6.75)	7.13 (4.97)	*p* = 0.506^1^	0.099^4^
AICA SNS score (m, SD)	8.02 (6.40)	7.73 (6.33)	8.60 (6.72)	*p* = 0.646^1^	0.068^4^
YSR					
Internalizing score (m, SD)	26.82 (11.67)	28.40 (11.22)	23.67 (12.30)	*p* = 0.242^1^	0.174^4^
Externalizing score (m, SD)	19.96 (10.33)	21.07 (10.25)	17.73 (10.48)	*p* = 0.462^1^	0.109^4^
FEEL-KJ					
Adaptive strategies (m, SD)	113.16 (29.42)	117.57(30.09)	104.33 (26.83)	*p* = 0.064^1^	0.276^4^
Adaptive strategies below norm (*n*, %)	20 (44.4)	10 (33.3)	10 (66.7)	*p* = 0.056^2^	0.316^3^
Adaptive strategies within norm (*n*, %)	23 (51.1)	19 (63.3)	4 (26.7)	*p* = 0.029^2^	0.346^3^
Adaptive strategies above norm (*n*, %)	2 (4.4)	1 (3.3)	1 (6.7)	*p* = 1.00^2^	0.076^3^
Maladaptive strategies (m, SD)	100.62 (15.76)	102.53(16.88)	96.80 (12.93)	*p* = 0.135^1^	0.222^4^
Maladaptive strategies below norm (*n*, %)	1 (2.2)	1 (3.3)	0 (0.0)	*p* = 1.00^2^	0.107^3^
Maladaptive strategies within norm (*n*, %)	11 (24.4)	6 (20.0)	5 (33.3)	*p* = 0.464^2^	0.146^3^
Maladaptive strategies above norm (*n*, %)	33 (73.3)	23 (76.7)	10 (66.7)	*p* = 0.496^2^	0.107^3^
PANAS (1 month prior to intervention)					
Positive emotions (m, SD)	15.82 (7.41)	16.43 (7.97)	14.60 (6.19)	*p* = 0.433^1^	0.116^4^
Negative emotions (m, SD)	20.00 (8.68)	20.27 (9.82)	19.43 (5.80)	*p* = 0.733^1^	0.065^4^
SEKJ (before intervention)					
Self-esteem Score (m, SD)	25.02 (10.20)	24.48 (10.85)	26.07 (9.09)	*p* = 0.519^1^	0.097^4^
Self-esteem below norm (*n*, %)	29 (65.9)	21 (72.4)	8 (53.3)	*p* = 0.315^2^	0.191^3^
Self-esteem within norm (*n*, %)	13 (29.5)	6 (20.7)	7 (46.7)	*p* = 0.092^2^	0.270^3^
Self-esteem above norm (*n*, %)	2 (4.5)	2 (6.9)	0 (0.0)	*p* = 0.540^2^	0.157^3^
Narrative Questions (at follow-up)					
Revisiting of the photos (*n*, %)	23 (51.1)	16 (53.3)	7 (46.7)	*p* = 0.303^2^	0.196^3^
Posting of the photos (*n*, %)	11 (24.4)	7 (23.3)	4 (26.7)	*p* = 1.000^2^	0.003^3^
Positive feelings toward photos (*n*, %)	26 (57.8)	15 (50.0)	11 (73.3)	*p* = 0.706^2^	0.113^3^
Ambivalent feelings toward photos (*n*, %)	6 (13.3)	4 (13.3)	2 (13.3)	p=1.000^2^	0.051^3^

According to the data, tests for gender differences were performed.

^1^ Mann–Whitney U-tests and ^2^ χ^2^-tests for categorical measures; m, mean; SD, standard deviation; CIUS, Compulsive Internet Use Scale; AICA, Assessment of Internet and Computer Game Addiction; YSR, Youth self-report; FEEL-KJ, Questionnaire for the evaluation of emotional regulation in children and adolescents; PANAS, Positive and Negative Affect Schedule; SEKJ, Self-worth inventory for children and adolescents; ^3^ Cramers V, interpretation according to Cohen (1988) 0.10 (small effect), 0.30 (medium effect), and 0.50 (large effect), ^4^ Cohen’s d, interpretation according to Cohen (1988) 0.20 (small effect), 0.50, (medium effect), and 0.80 (large effect).

### 3.2 Course of state emotions and SE over points of measurement

To tests for the difference in mean scores of variables assessed in repeated observations, a within-subject repeated-measures ANOVA was performed.

In girls, Mauchly’s test indicated that the assumption of sphericity had been violated for PANAS PE *χ* ^2^ *(9*) = 20.577, *p* = 0.015, and for PANAS NE *χ* ^2^ *(9*) = 18.586, *p* = 0.029, and therefore, a correction of degrees of freedom was done by using Greenhouse–Geisser estimates of sphericity (ε = 0.691 and ε = 0.724, respectively). A repeated-measures ANOVA with Greenhouse–Geisser correction determined that mean PANAS PE differed statistically significantly between time points [*F*(2.76, 66.30) = 17.83, *p* < 0.001], ω2 = 0.228 as well as PANAS NE [*F*(2.90, 69.54) = 34.15, *p* < 0.001], ω2 = 0.391. Mauchly’s test indicated that the assumption of sphericity had been met for SEKJ SE, *χ* ^2^ *(2*) = 0.855, *p* = 0.652. Multivariate tests showed a significant difference of SE means over all time points, *V* = 0.28, *F*(2, 22) = 4.342, *p* = 0.026, ω2 = 0.026.

In boys, Mauchly’s test indicated that the assumption of sphericity had been violated for PANAS NE, *χ* ^2^(9) = 18.026, *p* = 0.037, and therefore, a correction of degrees of freedom was done by using Greenhouse–Geisser estimates of sphericity (ε = 0.601). A repeated-measures ANOVA with Greenhouse–Geisser correction determined that mean PANAS NE differed statistically significantly between time points [*F*(2.40, 26.43) = 10.31, *p* < 0.001], ω2 = 0.318. Mauchly’s test indicated that the assumption of sphericity had been met for PANAS PE, *χ* ^2^(9) = 7.809, *p* = 0.558, and SEKJ SE, *χ* ^2^(2) = 3.669, *p* = 0.160. Multivariate tests showed that PANAS PE and SEKJ SE means differed statistically significantly over all time points, *V* = 0.678, *F*(4, 10) = 5.263, *p* = 0.015, ω2 = 0.070 and *V* = 0.469, *F*(2, 13) = 5.742, *p* = 0.016, ω2 = 0.020.

**Figure 2:**
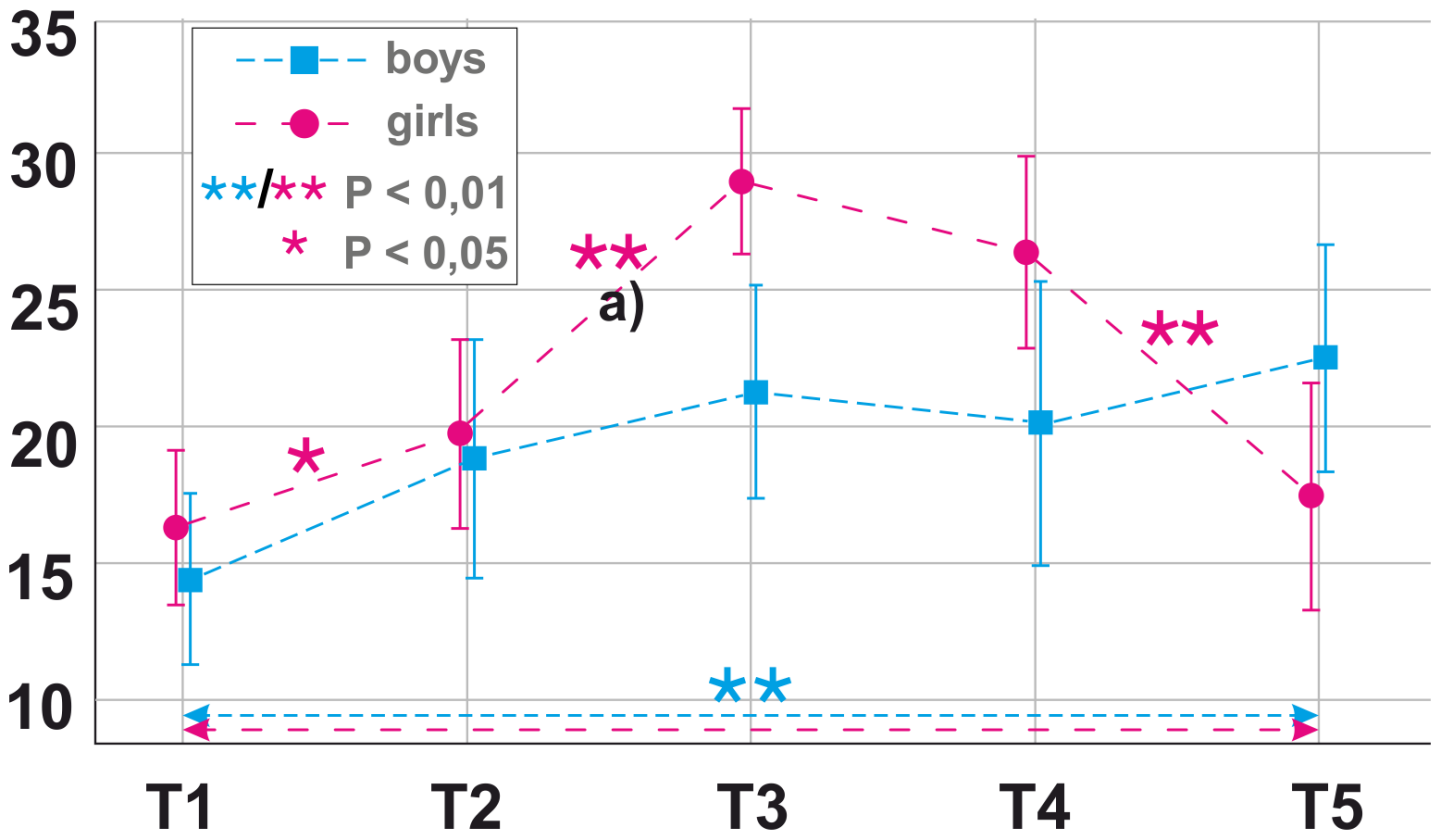
Longitudinal mean findings for positive state emotions (PE) as measured by Positive and Negative Affect Schedule (PANAS) over time by gender. T1 = 1 month prior to the photoshoot, T2 = immediately before the photoshoot, T3 = during the photoshoot, T4 = after the photoshoot/during receipt of photos, T5 = 3 months after the photoshoot/at follow-up. Significance [* < 0.5 level, ** < 0.01 level (two-tailed)] was calculated with paired-sample bootstrapped *t*-tests for difference in PE between consecutive time points. Alphabetic character **(A)** refers to significant linear regression model for adaptive strategies and internalizing problems in girls.

**Figure 3:**
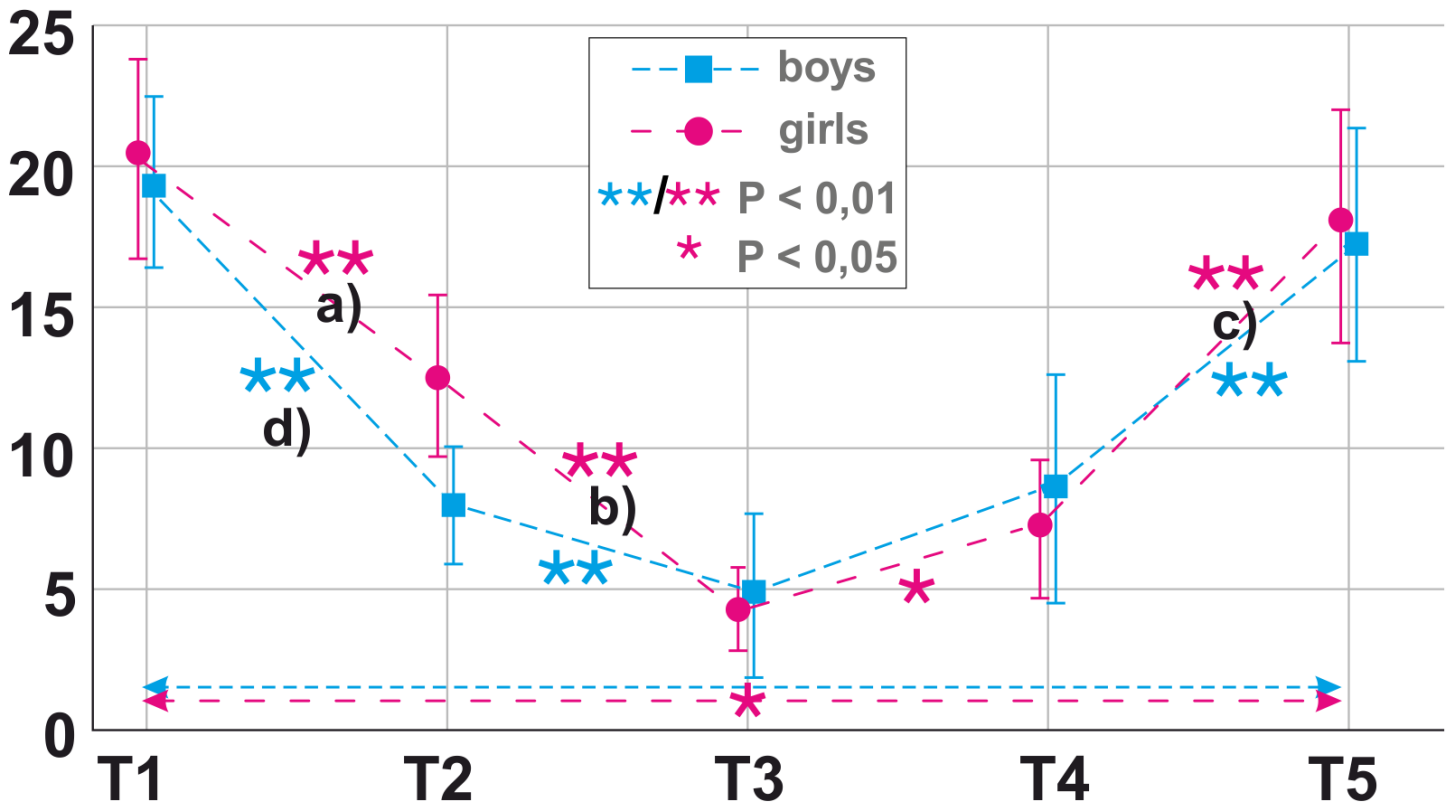
Longitudinal mean findings for negative state emotions (NE) as measured by Positive and Negative Affect Schedule (PANAS) over time by gender. T1 = 1 month prior to the photoshoot, T2 = immediately before the photoshoot, T3 = during the photoshoot, T4 = after the photoshoot/during receipt of photos, T5 = 3 months after the photoshoot/at follow-up. Significance [* < 0.5 level, ** < 0.1 level (two-tailed)] was calculated with paired-sample bootstrapped *t-*tests for difference in NE between consecutive time points. Alphabetic characters refer to significant linear regression models for **(A)** adaptive strategies; **(B)** internalizing problems, externalizing problems, Assessment of Internet and Computer Game Addiction (AICA) video streaming, and AICA online gaming; and **(C)** maladaptive strategies and externalizing problems in girls. Alphabetic characters refer to significant linear regression models for **(D)** maladaptive strategies in boys.

**Figure 4:**
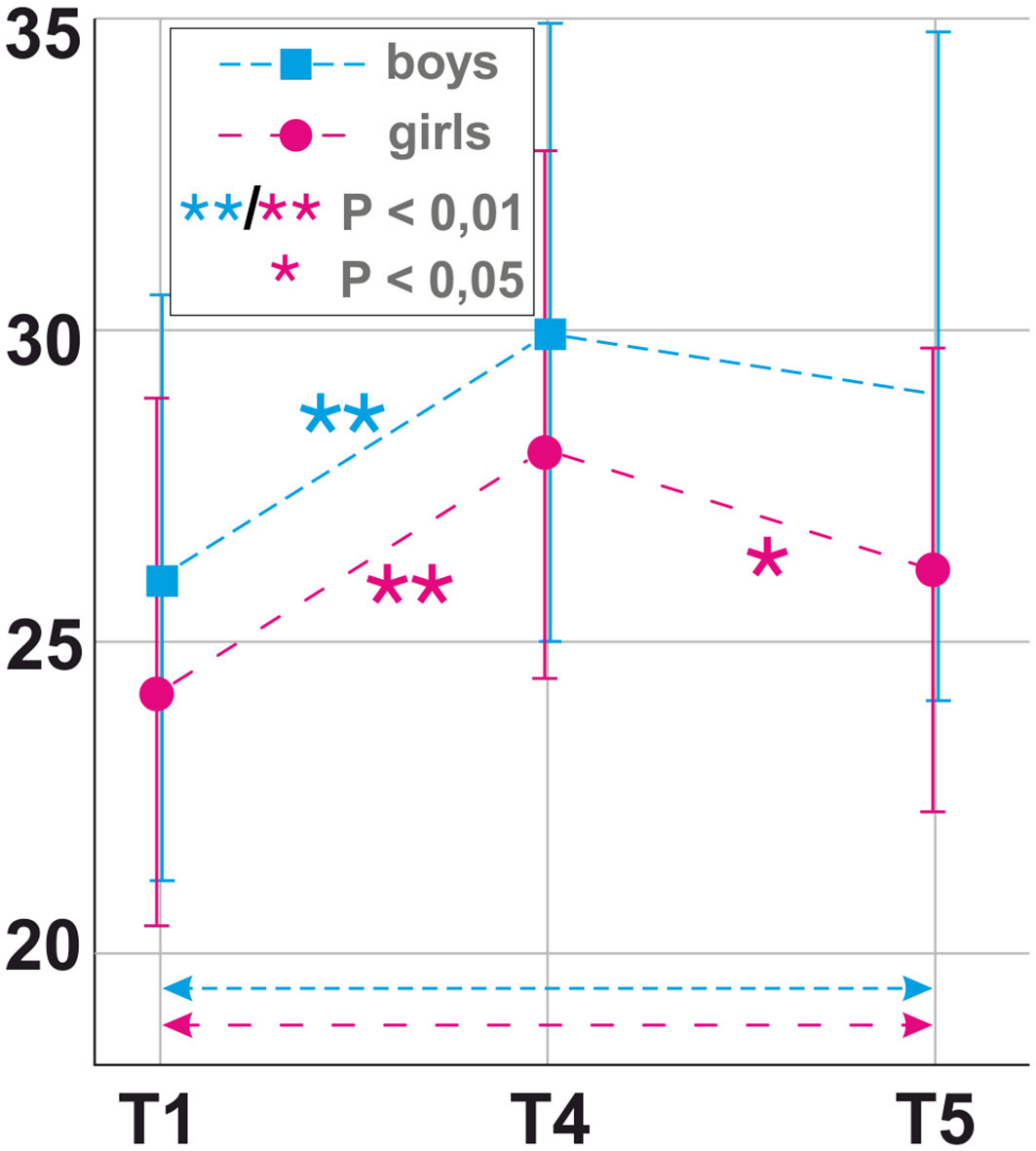
Longitudinal mean findings for self-esteem (SE) as measured by self-worth inventory for children and adolescents (SEKJ) over time by gender. T1 = 1 month prior to the photoshoot, T4 = after the photoshoot/during receipt of photos, T5 = 3 months after the photoshoot/at follow-up. Significance [* < 0.5 level, ** < 0.1 level (two-tailed)] was calculated with paired-sample bootstrapped *t-*tests for difference in SE between consecutive time points.

After significance of the interventional course was detected in within-subject repeated-measures ANOVA, bootstrapped paired-sample *t-*tests were performed to test for significance of mean changes in PE and NE as well as SE in both genders over the different time points of the course of the intervention (see **Figures 2**–**4**).

Girls showed a significant rise in PE between T1 (m = 16.48, SD = 8.38) and T2 (m = 19.92, SD = 10.26); *t*(24) = −2.171, *p* = 0.047 and T2 (m = 19.92, SD = 10.26) and T3 (m = 29.32, SD = 7.77); *t*(24) = −7.935, *p* < 0.001; as well as a significant decline between T4 (m = 26.60, SD = 10.10) and T5 (m = 17.64, SD = 10.92); *t*(24) = 4.837, *p* < 0.001 (**Figure 2**).

When looking at NE in girls, they highly significantly declined between T1 (m = 22.28, SD = 9.26) and T2 (m = 12.64, SD = 7.55); *t*(24) = 5.741, *p* < 0.001; T2 (m = 12.64, SD = 7.55) and T3 (m = 4.28, SD = 4.34); *t*(24) = 7.539, *p* < 0.001 and rose between T3 (m = 4.28, SD = 4.34) and T4 (m = 7.80, SD = 7.08); *t*(24) = −2.729, *p* = 0.013; T4 (m = 7.80, SD = 7.08) and T5 (m = 18.00, SD = 10.61); *t*(24) = −5.037, *p* < 0.001 (**Figure 3**). Nevertheless, an overall significant decline in NE was detected between T1 (m = 22.28, SD = 9.26) and T5 (m = 18.00, SD = 10.61); *t*(24) = 2.392, *p* = 0.028 (**Figure 3**). A highly significant rise in SE in girls was found between T1 (m = 25.13, SD = 11.39) and T4 (m = 30.00, SD = 11.14); *t*(23) = −2.939, *p* = 0.009; as well as a significant decline between T4 (m = 30.00, SD = 11.14) and T5 (m = 25.88, SD = 9.99); *t*(23) = 2.103, *p* = 0.042. (**Figure 4**).

Boys showed a highly significant rise in PE between T1 (m = 14.50, SD = 6.41) and T5 (m = 22.64, SD = 8.04); *t*(13) = −4.964, *p* = 0.005 (**Figure 2**). Regarding NE, a highly significant decline was found between T1 (m = 19.00, SD = 5.80) and T2 (m = 9.92, SD = 7.33); *t*(12) = 4.410, *p* = 0.004 and between T2 (m = 9.92, SD = 7.33) and T3 (m = 5.15, SD = 6.16); *t*(12) = 5.029, *p* = 0.001 as well as a rise between T4 (m = 9.15, SD = 8.45) and T5 (m = 17.31, SD = 8.23); *t*(12) = −3.105, *p* = 0.005 (**Figure 3**).

Concerning SE in boys, a highly significant rise was shown between T1 (m = 26.07, SD = 9.09) and T4 (m = 30.07, SD = 9.36); *t*(14) = −2.997, *p* = 0.010 (**Figure 4**).

### 3.3 Correlations

To assess possible influences of internet use-related variables, internalizing and externalizing symptomatology, and adaptive and maladaptive strategies on perceived emotions and SE during the different time points of the intervention, we performed two-tailed Spearman correlations. Results are shown in **Supplementary Table 1** and **Table 2**.

For YSR internalizing problems, girls showed significant negative correlations with PE at T1, T2, and T3 and significant positive correlations with NE at T1 and T2. In terms of FEEL-KJ adaptive strategies, significant positive correlations could be found with PE at T4 and T5 and significant negative correlations could be found with NE at T1 and T5. For FEEL-KJ maladaptive strategies, significant negative correlations could be found with PE at T1, T2, T3, and T5 and significant positive correlations could be found with NE at T1 (see **Supplementary Table 1**). Concerning possible influences on SE (SEKJ), girls showed significant negative correlations with CIUS total score, AICA SM use, AICA SNS use, YSR internalizing problems, and FEEL-KJ maladaptive strategies at receipt of the photos (T4). At baseline (T1), negative correlations were found with CIUS total score. At baseline (T1) and at follow-up (T5), significant negative correlations with AICA SNS, YSR internalizing problems, and FEEL-KJ maladaptive strategies were detected (see **Table 2**).

**Table 2:** Two-tailed Spearman correlations between self-esteem (SE) measured by SEKJ at the different time points with CIUS (general internet behavior), AICA (application-related behavior), YRS (internalizing and externalizing problems), and FEEL-KJ (coping strategies).

	m	SD	SEKJ		
			T1	T4	T5
Girls (*n* = 30)					
CIUS	22.2	9.8	−0.421*	−0.423*	−0.376
AICA CP	35.5	6.4	−0.290	−0.165	−0.261
AICA streaming	45.1	5.5	−0.048	−0.065	−0.168
AICA SM	42.9	6.7	−0.252	−0.399*	−0.291
AICA SNS	26.8	6.3	−0.449*	−0.537**	−0.431*
YSR internalizing	28.4	11.2	−0.694**	−0.407*	−0.406*
YSR externalizing	45.1	10.3	−0.256	−0.271	−0.419*
FEEL-KJ adaptive strategies	117.6	30.1	0.257	0.357	0.507**
FEEL-KJ maladaptive strategies	102.5	16.9	−0.662**	−0.507**	−0.622**
Boys (*n* = 15)					
CIUS	27.0	10.2	−0.022	−0.131	0.174
AICA CP	26.8	60.4	0.482	0.431	0.454
AICA streaming	24.7	6.1	0.346	0.178	0.559*
AICA SM	41.5	5.0	0.276	0.240	0.358
AICA SNS	22.1	6.7	0.301	0.214	0.458
YSR internalizing	23.7	12.3	−0.560*	−0.616*	−0.407
YSR externalizing	17.7	10.5	−0.032	−0.173	0.101
FEEL-KJ adaptive strategies	104.3	26.8	0.351	0.470	0.315
FEEL-KJ maladaptive strategies	96.8	12.9	−0.241	−0.247	−0.300

Correlation was performed for each gender separately.

** Correlation is significant at the 0.01 level (two-tailed), * Correlation is significant at the 0.05 level (two-tailed); m, mean; SD, standard deviation; CIUS, Compulsive Internet Use Scale; AICA, Assessment of Internet and Computer Game Addiction (CP, computer gaming; Stream, video streaming; SM, social media; SNS, social networking sites); YSR, Youth self-report (Int, internalizing problems; Ext, externalizing problems); FEEL-KJ, questionnaire for the evaluation of emotional regulation in children and adolescents; SEKJ, self-worth inventory for children and adolescents; T1 = 1 month prior to the photoshoot; T4, after the photoshoot/during receipt of photos; T5 = 3 months after the photoshoot/at follow-up.

Boys showed significant positive correlations between PE at receipt of the photos (T4) with AICA video streaming, AICA SNS, and AICA SM. Furthermore, significant positive correlations between FEEL-KJ maladaptive strategies with NE at T1 and T5 could be found (see **Supplementary Table 1**). When assessing possible influences on SE (SEKJ) in boys, significant negative correlations with YSR internalizing problems at baseline (T1) and receipt of the photos (T4) were found. Furthermore, significant positive correlations were detected between AICA streaming and SEKJ T5 (see **Table 2**).

### 3.4 Simple linear regression

For those sections in the interventional course (**Figures 2**–**4**; PANAS, SEKJ) that showed a significant rise or decline, simple linear regressions were administered to test if individual variables significantly influence the section history. We used the following independent variables: internalizing problems (YSR), externalizing problems (YSR), adaptive strategies (FEEL-KJ), maladaptive strategies (FEEL-KJ), internet use (CIUS), and specific internet use (AICA).

In girls, several significant regression equations were found: Adaptive strategies significantly negatively predicted [*R* ^2^ of 0.26, *F*(1,28) = 9.69, *p* = 0.004, *t*(28) = −3.11, *β* = −0.10] and internalizing problems significantly positively predicted rise in PE from T2 to T3 [*R* ^2^ = 0.20, *F*(1,28) = 7.036, *p* = 0.013, *t*(28) = 2.653, *β* = 0.226] (see **Figure 2A**).

Adaptive strategies positively predicted decline in NE from T1 to T2 [*R* ^2^ = 0.18, *F*(1,28) = 5.993, *p* = 0.021, *t*(28) = 2.448, *β* = 0.117] (see **Figure 3A**). Internalizing problems [*R* ^2^ = 0.16, *F*(1,28) = 5.379, *p* = 0.028, *t*(28) = −2.319, *β* = −0.205], externalizing problems [*R* ^2^ = 0.16, *F*(1,28) = 5.499, *p* = 0.026, *t*(28) = −2.345, *β* = −0.226], AICA-video streaming [*R* ^2^ = 0.14, *F*(1,28) = 4.372, *p* = 0.046, *t*(28) = −2.091, *β* = −0.385], and AICA-computer gaming [*R* ^2^ = 0.20, *F*(1,28) = 6.813, *p* = 0.014, *t*(28) = −2.610, *β* = −0.396] negatively predicted decline in NE from T2 to T3 (see **Figure 3B**). The rise in NE from T4 to T5 was positively predicted by FEEL-KJ maladaptive strategies [*R* ^2^ of 0.23, *F*(1,23) = 6.90, *p* = 0.015, *t*(23) = 2.63, *β* = 0.28] and externalizing problems [*R* ^2^ = 0.20, *F*(1, 23) = 5.638, *p* = 0.026, *t*(23) = 2.374, *β* = 0.429] (see **Figure 3C**).

For boys, only one regression equation was detected: Maladaptive strategies significantly negatively predicted the decline in NE from T1 to T2 [*R* ^2^ = 0.36, *F*(1,11) = 6.12, *p* = 0.031, *t*(11) = −2.47, *p* = 0.031. *β* = −0.34] (see **Figure 3D**).

### 3.5 Narrative follow-up on assessment

Seventy-three percent of boys and 50% of the girls reported positive feelings towards the photos. Fifty-one percent of the participants reported revising the photos, and 24% of the sample posted the photos online. For further details, see **Table 1**.

## 4 Discussion

Through the use of SM and SNS, social comparison with idealized young people, especially in terms of appearance, is omnipresent among adolescents (57, 58). Mediated by the use of photo editing, the perception of the average external appearance diverges from the actual reality of the norm (e.g., thin, toned, well-exercised, healthy eating, and weight loss-encouraging individuals) (59, 60). This perceptual shift can lead to negative influence on mental health, such as body (61) and face dissatisfaction (62). In the study presented, we developed a new approach to CAP patients by offering a professional PS in the treatment setting. We evaluated the outcome on SE and state emotions at different points of the intervention (in anticipation of the PS, during the PS, at receiving the photos, and long-term outcome).

Internet use in our population was problematic (PIU) in 50% of girls and 80% of boys. In girls, the results are consistent with clinical populations; in boys, they exceed the previously reported rates (63, 64). A possible explanation might be an increase in prevalence of PIU during the COVID pandemic (65, 66). Compared to the test norm, our subjects showed increased maladaptive defense strategies and reduced adaptive ones, as well as reduced SE, especially the girls.

During the photo intervention, substantial fluctuations in emotionality and SE could be detected. Girls reported a significant rise in PE and SE and a decline in NE from baseline to the PS, possibly caused by anticipation. Being invited to a PS was possibly associated with good expectations, receiving centered attention and the photo-shooting atmosphere might have enhanced wellbeing. In girls, the positive emotional effect and increase in SE, however, could not be sustained after receiving the pictures. In boys, overall, in terms of positive emotionality and SE, the intervention had a beneficial outcome. Reacting less enthusiastically in the expectation phase from baseline to PS, an overall outcome of heightened positive emotions at follow-up could be observed. SE significantly rose and remained at higher levels. Only NE over the course of the intervention were similar to those in the girls.

In general, adolescents tend to experience high-intensity emotions and higher emotional instability compared to children and adults (67, 68). The gender differences in affect converge with studies reporting elevated emotional reactivity in female when compared with male adolescents (69–74), especially in terms of negative affect (71–74). A possible explanation might be higher probability of negative life events and increased cognitive vulnerability in girls (72). In addition, adolescent boys have been shown to have more efficient regulatory mechanisms for negative affect as well as greater hedonic balance levels (75).

### 4.1 The role of defense strategies in relation to the intervention

In girls, coping mechanisms were relevant for the emotional perception and experienced SE at different time points of the intervention. Maladaptive strategies diminished PE before and during the shooting and mediated a rise in NE after the intervention and were associated with low levels of SE throughout the intervention. Adaptive strategies lowered positive expectations before the intervention and, at the same time, relevantly diminished NE and allowed PE to remain higher after the intervention. High levels of adaptive strategies allowed SE to remain elevated after the intervention. No significant influence on the course of the intervention due to coping strategies was found in boys.

Hence, our results reflect a remarkable gender difference in emotion regulation and SE as a function of coping with an interventional offer. In adolescence, a decreased use of adaptive strategies and an increased use of maladaptive strategies have been described (76, 77). There is consensus that in stressful situations, different coping strategies are more likely to be used within the genders, respectively (78–80). Especially in girls, a stronger decrease in humor enhancement and cognitive problem solving ability has been reported (77). The higher vulnerability of girls to mental illness in adolescence can therefore also be interpreted as a function of limited defense strategies (81, 82).

### 4.2 The role of psychopathological symptoms in relation to the intervention

Girls with internalizing symptoms experienced a lower baseline but a significantly higher rise in positivity and decline in negativity during the intervention. This emotional uplift triggered by the intervention could be specifically therapeutically used in internalizing patients, for instance, as index moment in cognitive behavioral therapy. Furthermore, internalizing symptoms were associated with lower SE throughout the intervention in both genders. Internalizing problems are defined as being directed inwardly and express internal distress (83). They include symptoms related to depression and anxiety and lower SE (84–86). Female adolescents are more likely to experience depression compared to male adolescents and prevalence rates are constantly rising (72, 87). Externalizing symptoms were found to be associated with a rise in negative emotions after the intervention in girls. Externalizing problems include symptoms related to aggression, reduced reward dependence, hostile affect, and delinquency (86, 88). Prevalence rates of externalizing problems such as deviant behaviors and conduct behaviors are increasingly relevant in girls, while prevalence rates for this diagnostic category decreased in male adolescents over time (89, 90).

### 4.3 The role of internet-related variables in relation to the intervention

High levels of SNS use and internet use in girls were associated with low levels of SE during the intervention. SM use could be linked to low SE when being confronted with the pictures. Interestingly, this negative outcome in relation to SNS or SM could not be observed in boys. In contrast, the use of streaming, SM and SNS lead to elevated positive emotion at receipt of the photos.

In general, problematic mobile phone and SM/SNS use is more prevalent among female adolescents (91, 92), whereas among male adolescents, there are higher prevalence rates in gambling and online gaming disorders (92). Social comparison, social feedback, and self-reflection are the three key mechanisms contributing to a possible relation between SM/SNS and SE (93). Building up SE is an important developmental task during adolescence and a predictor of later psychological wellbeing (94, 95), healthy peer attachment (96), and success in life (94). SE in female adolescents was found to be more social-oriented and reward-oriented compared to their male counterparts (80, 97–99). In accordance, female adolescents reported more often to benefit from the use of SM/SNS in order to satisfy their social needs (100, 101). On the other hand, social expectations can promote problematic SM/SNS use (102, 103). When it comes to boys, researchers have found that they show a more robust SE (75). Male adolescents show less concern with feelings of others due to a higher sensitivity threshold to others’ emotions (104). Male adolescents tend to be more involved in competitive internet activities as represented by online gaming (105, 106).

Adolescent girls are more exposed to stressors such as concerns about their body image (107). Tendency to self-objectification, to monitor attractive individuals (108), and to carry out social comparison on SM was found to be related to lower SE (109). The marketing-driven SM industry is geared towards upward social comparison (comparing oneself to others who are better off) (110, 111) and judgmental body image (112), leading to dissatisfaction, lower levels of SE, and emotional distress (113, 114). In particular, the algorithm-driven passive form of SM use is reported to be related to increased social comparison (115, 116), lower wellbeing (57), body dissatisfaction (115), and depression (117).

### 4.4 Limitations and strengths

The focus of our study was to assess the impact of a new intervention, namely, a PS, on adolescents undergoing psychiatric treatment. We were interested in examining the different time points of the intervention to determine how patients react at different stages of the intervention and whether certain factors such as internet use, coping mechanisms, or psychopathology had an impact on the course. However, our specific approach was associated with limitations: Firstly, our sample included significantly more girls, which, at the same time, reflects the reality of gender distribution of adolescents in psychiatric treatment (118). However, the unequal gender ratio and the smaller sample size of boys must be taken into account when interpreting the results. It is possible that detection of statistically relevant effects was more unlikely in the male sample. In general, the sample size was rather small, but it should be borne in mind that the interventional study design was rather elaborate. Another limitation is that our study took place in CAP patients with an expected ceiling effect in psychopathology as well as negative coping and SE. Therefore, the results cannot be generalized to non-clinical adolescents and future studies including a non-clinical comparison group would be of relevance. In particular, the negative effects of internet and SNS/SM use on the outcome of the intervention in girls could be related to the specific vulnerability of our sample.

One of the strengths of the study is the new approach to working with young people with psychiatric disorder. The study was met with great interest and willingness on the part of the young people to participate. This is also reflected in the fact that all of the young people who took part stayed on for the entire intervention and the majority of the participants (*n* = 40) remained accessible for the follow-up study. Three months after receiving the photos, 73% of the boys rated their photos positively and 63.3% of the girls rated them positively or at least ambivalently. Professional camera equipment and instruction led to higher-quality portrait photography compared to (self-guided) mobile phone camera use. Using a professional camera instead of a mobile phone camera demarcated the intervention from the topic of SNS and SM. The results converge with the literature and substantially add to our knowledge on new treatment approaches in CAP patients.

### 4.5 Conclusion

The novel intervention using PS was well received by patients. Girls experienced a positive development in SE and an increase in positive emotion, particularly in anticipation of and during the PS. Perhaps the offer represents a contrast to other interventions because it provides centered attention and focus while maintaining intimacy. Increase in PE and SE experienced could be used and processed therapeutically. Furthermore, our results add to our understanding on the relevance of coping mechanisms in the processing of offers in CAP patients. Our results suggest that it is relevant to reflect on SM behavior with girls in terms of the impact on their SE. For boys, the intervention appears to be largely beneficial. The use of SM and SNS does not seem to have a negative influence on their perception of their individual photos. Although practitioners are generally less likely to consider that interventions relating to external attributes might be relevant for boys, this does appear to be the case.

## Data availability statement

The raw data supporting the conclusions of this article will be made available by the authors, without undue reservation.

## Ethics statement

The studies involving humans were approved by Ethics committee of the state of Salzburg (ethic committee vote-number: 1091/2021). The studies were conducted in accordance with the local legislation and institutional requirements. Written informed consent for participation in this study was provided by the participants’ legal guardians/next of kin.

## Author contributions

KW: Data curation, Formal Analysis, Investigation, Methodology, Project administration, Software, Visualization, Writing – original draft, Writing – review & editing. TM: Conceptualization, Project administration, Writing – review & editing. BS: Visualization, Writing – review & editing, Formal Analysis, Supervision. NR: Data curation, Project administration, Writing – review & editing. CS: Formal Analysis, Writing – review & editing, Methodology. MH: Conceptualization, Project administration, Writing – review & editing. JT-S: Conceptualization, Writing – review & editing, Project administration. LR: Formal Analysis, Methodology, Writing – review & editing, Data curation. WH: Writing – review & editing, Formal Analysis, Methodology. CA: Supervision, Writing – review & editing, Validation. BP: Conceptualization, Data curation, Formal Analysis, Methodology, Project administration, Supervision, Visualization, Writing – original draft, Writing – review & editing.
