# Supplementary material for: Glow up: does a professional photoshoot intervention affect self-esteem and emotions among adolescent psychiatric patients?—A longitudinal intervention study
Source: Front Psychiatry. 2024 Feb 23;15:1310252. doi: 10.3389/fpsyt.2024.1310252 (PMC10920346; doi:10.3389/fpsyt.2024.1310252)
Supplement: Supplementary file 1 [file Table_1.docx]

Supplementary Material

# Supplementary Table

**Supplementary Table 1**. Two tailed Spearman correlations between positive and negative state emotions (PE/NE) measured by PANAS at the different timepoints with CIUS (general internet behavior), AICA (application related behavior), YRS (internalizing and externalizing problems), FEEL-KJ (coping strategies). Correlation was performed for each gender separately.

|  |  |  | PANAS |  |  |  |  |  |  |  |  |  |
| --- | --- | --- | --- | --- | --- | --- | --- | --- | --- | --- | --- | --- |
|  | m | SD | PE T1 | PE T2 | PE T3 | PE T4 | PE T5 | NE T1 | NE T2 | NE T3 | NE T4 | NE T5 |
| Girls (n=30) |  |  |  |  |  |  |  |  |  |  |  |  |
| CIUS | 22,2 | 9,8 | -.143 | -.096 | -.030 | -.218 | -.158 | .127 | .039 | .201 | .132 | .416^*^ |
| AICA CP | 35,5 | 6,4 | -.089 | .046 | .111 | .058 | -.084 | .138 | .167 | -.031 | -.032 | .143 |
| AICA Streaming | 45,1 | 5,5 | .017 | -.028 | .023 | .064 | .071 | .179 | -.063 | -.261 | -.223 | .114 |
| AICA SM | 42,9 | 6,7 | -.243 | -.287 | -.191 | -.171 | -.139 | .136 | .012 | .028 | -.024 | .194 |
| AICA SNS | 26,8 | 6,3 | -.228 | -.261 | -.134 | -.228 | -.198 | .114 | .002 | .102 | .001 | .358 |
| YSR Internalizing | 28,4 | 11,2 | -.515^**^ | -.502^**^ | -.491^**^ | -.093 | -.267 | .563^**^ | .512^**^ | .128 | -.002 | .319 |
| YSR Externalizing | 45,1 | 10,3 | -.087 | -.146 | -.059 | -.213 | -.505^*^ | .274 | .206 | .065 | .065 | .493^*^ |
| FEEL-KJ adaptive strategies | 117,6 | 30,1 | .283 | .328 | .183 | .378^*^ | .529^**^ | -.481^**^ | -.271 | -.142 | -.280 | -.404^*^ |
| FEEL-KJ maladaptive strategies | 102,5 | 16,9 | -.450^*^ | -.372^*^ | -.412^*^ | -.168 | -.547^**^ | .387^*^ | .309 | .087 | .091 | .375 |
| Boys (n=15) |  |  |  |  |  |  |  |  |  |  |  |  |
| CIUS | 27,0 | 10,2 | -.107 | .278 | .575^*^ | .343 | .181 | .122 | -.282 | -.117 | -.032 | .122 |
| AICA CP | 26,8 | 60,4 | .347 | .079 | .300 | .427 | .251 | .020 | .288 | .007 | -.034 | .292 |
| AICA Streaming | 24,7 | 6,1 | .131 | .228 | .412 | .643^**^ | .319 | .339 | .134 | .151 | -.136 | .069 |
| AICA SM | 41,5 | 5,0 | .145 | -.115 | .294 | .535^*^ | .296 | .082 | .298 | .182 | -.169 | .268 |
| AICA SNS | 22,1 | 6,7 | .220 | .053 | .315 | .578^*^ | .323 | .171 | .141 | .007 | -.203 | .205 |
| YSR Internalizing | 23,7 | 12,3 | -.506 | -.236 | -.059 | -.170 | -.282 | .453 | -.120 | .241 | .332 | .244 |
| YSR Externalizing | 17,7 | 10,5 | .223 | .177 | .144 | .117 | .235 | .033 | -.065 | .020 | .070 | .258 |
| FEEL-KJ adaptive strategies | 104,3 | 26,8 | .615^*^ | .023 | -.057 | .220 | .399 | .071 | .095 | -.176 | -.332 | -.286 |
| FEEL-KJ maladaptive strategies | 96,8 | 12,9 | .085 | .104 | .027 | .006 | -.072 | .581^*^ | .164 | .154 | .380 | .555^*^ |
| **Note:** ** Correlation is significant at the .01 level (2-tailed), * Correlation is significant at the .05 level (2-tailed); m= mean, SD= standard deviation, CIUS= Compulsive Internet Use Scale, AICA= Assessment of Internet and Computer Game Addiction (CP= Computer gaming, Stream = Video streaming, SM= Social Media, SNS= Social Networking Sites), YSR= Youth self report (Int= Internalizing problems, Ext= Exteernalizing problems), FEEL-KJ = Questionnaire for the evaluation of emotional regulation in children and adolescents, PANAS= Positive and Negative Affect Schedule: PE= Positive State Emotions, NE= Negative State Emotions; T1= one month prior to photoshoot, T2= immediately before photoshoot, T3= during photoshoot, T4= after photoshoot/during receipt of photos, T5= three month after photoshoot/at follow-up | | | | | | | | | | | | |

# Results

## Post-hoc analysis for within-subject repeated measure ANOVA

In girls post hoc analysis with Bonferoni adjustment revealed that PANAS PE was significantly increased from T2 (M= 19.92, SD= 10.255) to T3 (M= 29.32, SD= 7.766), p< .001 as well as significantly decreased from T4 (M= 26.60, SD= 10.100) to T5 (M= 17.64, SD= 10.924), p< .001. PANAS NE was significantly decreased from T1 (M= 22.28, SD= 9.258) to T2 (M= 12.64, SD= 7.549), p< .001 as well as T2 (M= 12.64, SD= 7.549) to T3 (M= 4.28, SD= 4.335), p < .001 and significantly increased from T4 (M= 7.80, SD= 7.077) to T5 (M= 18.00, SD= 10.607), p< .001. SEEKI SE was significantly increased from T1 (M= 25.12, SD= 11.388) to T2 (M= 30.00, SD= 11.136), p= .022.

In boys post hoc analysis with Bonferoni adjustment revealed that PANAS PE was significantly increased from T1 (M= 14.50, SD= 6.406) to T5 (M= 22.64, SD= 8.044), p= .003. PANAS NE was significantly decreased from T1 (M= 19.67, SD= 5.516) to T2 (M= 9.92, SD= 7.657), p< .008 as well as T2 (M= 9.92, SD= 7.657) to T3 (M= 5.58, SD= 6.230), p= .006. SEEKI SE was significantly increased from T1 (M= 26.07, SD= 9.091) to T2 (M= 30.07, SD= 9.362), p= .029.

## Course of state emotions and SE over points of measurement according to type of care

When grouping by type of care (inpatient, outpatient, daily clinic) Mauchly’s test indicated that the assumption of sphericity had been met for PANAS NE male inpatients X^2^(9)= 15.135, p= .107. Multivariate tests showed that PANAS NE means differed statistically significantly over all time points, V= .941, F(4, 3)= 11.938, p= .035, ω^2^= .400.

In girls Mauchly’s test indicated that the assumption of sphericity had been violated for PANAS PE inpatients X^2^(9)= 24.604, p= .004 as well as PANAS PE outpatients X^2^(9)= 23.165, p= .007 and therefore a correction of degrees of freedom was done by using Greenhouse-Geisser estimates of sphericity (ε= .378 and ε= .480, respectively). A repeated measures ANOVA with Greenhouse-Geisser correction determined that mean PANAS PE inpatients (F(1.51, 13.61)= 9.32, p= .005, ω^2^= .300) as well as PANAS PE outpatients (F(1.92, 15.35)= 4.785, p= .025, ω^2^= .211) differed statistically significantly between time points). Mauchly’s test indicated that the assumption of sphericity had been met for PANAS NE inpatients X^2^(9)= 12.382, p= .203.Multivariate tests showed that PANAS NE inpatients means differed statistically significantly over all time points, V= .876, F(4, 6)= 10.644, p= .007, ω^2^= .392.

To measure further mean changes in PE and NE as well as SE in both genders over the course of the intervention bootstrapped according to the type of care (inpatient, outpatient, daily-clinic) paired sample t-test were performed. The inpatient sample consisted of 17 participants (11 females), outpatient sample of 13 participants (9 females), and the daily clinic of a 8 participants (6 females).

The results indicated that among female participants undergoing inpatient treatment there were significant differences 1) between PANAS PE T1 (m=11.70, SD = 6.550) and T2 (m= 15.90, SD= 8.252), t(9) = -3.194, p= .011 2) between PANAS PE T2 (m= 15.90; SD= 8.252) and T3 (m= 26.90, SD= 7.520), t(9) = -6.398, p= <.001 3) between PANAS PE T4 (m= 25.40, SD= 10.211) and T5 (m= 15.10, SD= 10.867), t(9) = 4.057, p = .003 4) between PANAS NE T1 (m= 26.50, SD = 5.543) and T2 (m= 15.70, SD= 7.334), t(9)= 4.227, p= .002 5) between PANAS NE T2 (m= 15.70, SD= 7.334) and T3 (m= 6.60, SD= 5.562), t(9)= 5.609, p <.001 6) between PANAS NE T4 (m= 8.90, SD= 6.008) and T5 (m= 22.30, SD= 9.557), t(9)= -5.499, p <.001 7) between SEKJ T1 (m= 15.60, SD= 4.169) and T4 (m= 22.80, SD= 10.130), t(9)= -2.502, p= .034

Furthermore, among female participants undergoing outpatient treatment there were significant differences 1) between PANAS PE T1 (m= 23.11, SD= 5.732) and T2 (m= 28.67, SD= 6.928), t(8)= -5.793, p= <.001 2) PANAS PE T2 (m= 28.67, SD = 6.928) and T3 (m= 34.33, SD = 5.050), t(8)= -4.185, p= .003 3) PANAS NE T2 (m= 10.22, SD= 8.363) and T3 (m= 3.00, SD= 2.646), t(8)= 3.146, p= .014 4) PANAS NE T3 (m= 3.00, SD= 2.646) and T4 (m= 9.11, SD= 9.266), t(8)= -2.498, p= .037

Among female participants undergoing daily-clinic treatment there were significant differences 1) between PANAS PE T2 (m= 13.50, SD= 9.268) and T3 (m= 25.83, SD = 8.681), t(5)= -4.348, p= .007 2) between PANAS PE T4 (m= 24.50, SD= 10.913) and T5 (m= 10.00, SD= 4.427), t(5)= 3.738, p= .013 3) between PANAS NE T1 (m= 27.33, SD= 6.743) and T2 (m= 11.17, SD= 5.742), t(5)= 6.248, p= .002 4) between PANAS NE T2 (m= 11.17, SD= 5.742) and T3 (m= 2.33, SD= 2.251), t(5)= 4.771, p= .005 5) between PANAS NE T4 (m= 4.00, SD= 3.899) and T5 (m= 21.00, SD= 8.672), t(5)= -5.020, p= .004 6) between SEKJ T1 (m= 24.80, SD= 3.899) and T4 (m= 32.80, SD= 6.458), t(4)= -2.902, p= .044 7) between SEKJ T4 (m= 32.80, SD= 6.458) and T5 (m= 22.40, SD= 3.647), t(4)= 2.856, p= .046

Among male participants undergoing inpatient treatment there were significant differences 1) between PANAS PE T1 (m= 12.63, SD= 7.050) and T5 (m= 19.38, SD= 7.909), t(7)= -2.899, p= .023 2) between PANAS NE T1 (m= 18.57, SD= 5.563) and T2 (m= 9.14, SD= 4.670), t(6)= 4.032, p= .007 3) between PANAS NE T2 (m= 9.14, SD= 4.670) and T3 (m= 4.57, SD= 4.353), t(6)= 4.701, p= .003 4) between PANAS NE T4 (m= 12.00, SD= 6.733) and T5 (m= 20.29, SD= 8.712), t(6)= -2.848, p= .029 5) between SEKJ T1 (m= 21.44, SD= 8.002) and T4 (m= 26.22, SD= 9.148), t(8)= -2.724, p= .026 6) between SEKJ T1 (m= 21.44, SD= 8.002) and T5 (m= 25.00, SD= 8.703), t(8)= -2.340, p= .047

Among male participants undergoing outpatient treatment there were significant differences 1) between PANAS PE T1 (m= 16.75, SD= 5.620) and T5 (m= 26.25, SD= 7.588), t(3)= -4.701, p= .018 2) between PANAS NE T1 (m= 16.75, SD= 5.620) and T5 (m= 26.25, SD= 7.588), t(2)= 5.270, p= .034.

**
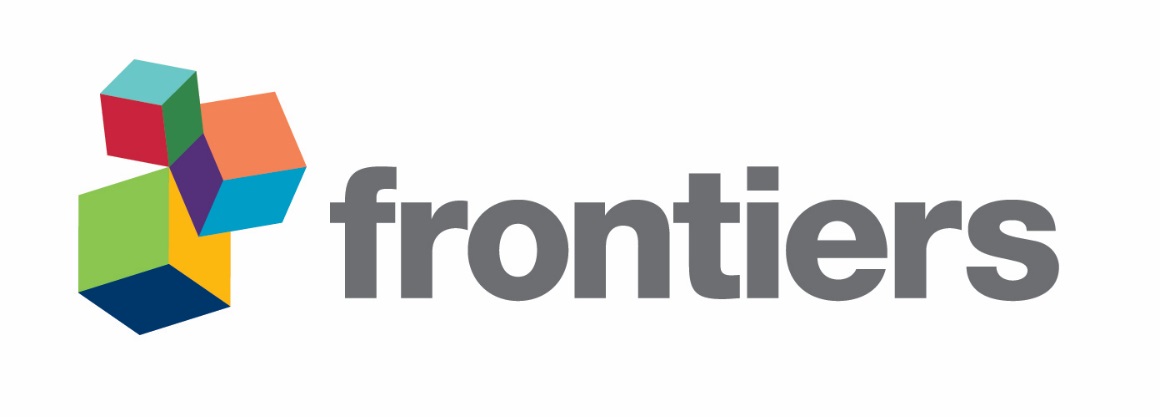
**
